# Photovoice: A Novel Approach to Improving Antituberculosis Treatment Adherence in Pune, India 

**DOI:** 10.1155/2014/302601

**Published:** 2014-10-07

**Authors:** Sangita C. Shelke, Prakash S. Adhav, Patrick K. Moonan, Matthew Willis, Malan A. Parande, Srinath Satyanarayana, Vikas D. Kshirsagar, Smita Ghosh

**Affiliations:** ^1^Department of Preventive and Social Medicine, Byramjee Jeejeebhoy Government Medical College, Pune, India; ^2^Division of Tuberculosis Elimination, Centers for Disease Control and Prevention, Atlanta, GA, USA; ^3^International Union Against Tuberculosis and Lung Disease, South-East Asia Regional Office, New Delhi, India

## Abstract

We compared antituberculosis treatment (ATT) adherence and outcomes among patients exposed to Photovoice (video of previously cured TB patients sharing experiences about TB treatment) versus those not exposed. The odds of successful outcome (i.e., cured or completing treatment) for the 135 patients who watched Photovoice were 3 times greater (odds ratio: 2.8; 95% CI: 1.3–6.1) than for patients who did not watch Photovoice. The comparison group, on average, missed more doses (10.9 doses; 95% CI: 6.6–11.1) than the intervention group who saw Photovoice (5.5 doses; 95% CI: 3.7–6.1). Using Photovoice at initiation of ATT has the potential to improve treatment adherence and outcomes.

## 1. Introduction

Tuberculosis (TB) requires successful completion of at least six months of antituberculosis treatment (ATT) [1]. Unfortunately, ATT compliance is difficult. Long treatment duration, common side effects of the medication, and other problems such as poverty and unemployment have been reported as potential barriers to ATT adherence [2]. ATT nonadherence leads to prolonged infectiousness, acquisition of drug resistance, relapse, lower cure rates, and higher mortality rates [3, 4]. In 2012, more than a third of the states in India reported that >20% of patients either lost to follow-up or were not cured at the conclusion of ATT [5], suggesting that a substantial proportion of patients are nonadherent to ATT.

ATT adherence often depends upon the patients' general knowledge of TB, socioeconomic status, and whether they believe in the efficacy of the medication [6, 7]. Photovoice is an anthropologic storytelling method that allows participants to use mixed media to record and share personal experience to influence behavioural change and promote public health action [8]. In our study, we used video recordings to capture the personal experiences about tuberculosis treatment and treatment adherence of previously treated tuberculosis patients. We sought to determine if watching a Photovoice video before ATT would reduce the number of missed doses and improve treatment outcomes.

## 2. Methods

The study was conducted at BJGMC, Sassoon General Hospital, Government TB Treatment Centre. BJGMC treats approximately 4,000 tuberculosis patients per year and offers medical service to over 9 million people of the Pune, the second most populous district in Maharashtra state [9]. Utilizing the Photovoice approach [7], a 15-minute video was produced in Marathi, the local language spoken in Maharashtra. The video production, costing approximately $500, included eight previously cured patients expressing their personal stories about TB, their attitudes, perceptions and beliefs about ATT, and how ATT had a positive effect on their well-being and recovery from illness. All patients emphasized the benefits of treatment adherence and importance of completing ATT. We approached every third newly registered patient at BJGMC during January 1, 2011, to March 31, 2011, to participate in the study. After giving written informed consent, patients were shown the Photovoice video and initiated ATT as per national guidelines [5]. Every third treatment card of newly registered patient during January 1, 2010, to March 31, 2010, was reviewed as a comparison group. These patients had not viewed the Photovoice video. No other interaction with study investigators occurred after the start of treatment for both groups. New TB patients were defined as persons not previously treated for TB and in the first month of ATT. Previously treated patients were not approached to enroll nor were they included in the comparison group. After TB diagnosis all patients were referred to a Directly Observed Treatment Strategy (DOTS) centre located nearest to their residence. Each DOTS centre administers ATT and maintains a TB treatment card documenting the number of missed doses during TB treatment and treatment outcomes upon completion.

Demographic and clinical variables, number of doses prescribed, number of doses taken during treatment, and treatment outcomes were collected from the TB treatment cards of patients in both the Photovoice and comparison groups. Treatment outcomes were defined as successful or unsuccessful according to national treatment guidelines described elsewhere [5]. Successful treatment outcome included patients who were considered cured or who completed a full course of ATT. Unsuccessful treatment outcome included patients who failed treatment, transferred, lost to follow-up, or died during ATT [5].

Pearson chi-square and independent sample *t*-tests were used to compare differences in proportions between successful and unsuccessful outcome among the Photovoice and comparison groups. Odds ratios (OR) were used to measure the association of Photovoice with sputum status at the end of the intensive phase (IP) and with treatment outcomes. All statistical tests were considered to be significant at an alpha of <0.05.

All patients were treated according to national guidelines irrespective of their participation or refusal to take part in the study. All data were safeguarded to protect patient confidentiality and no individual patient identifiers were retained electronically. The study protocol was approved by the International Union Against TB and Lung Disease (Paris, France) and the Institutional Review Board of BJGMC.

## 3. Results

Among 144 patients selected to view the Photovoice video, 135 (94%) consented to participate. In total, 276 patients (Photovoice group *n* = 135; comparison group *n* = 141) were studied. There were no significant differences in age, HIV status, or clinical presentation between Photovoice and comparison groups (Table 1). Almost two-thirds (76%) of the patients who watched the Photovoice video did not miss any doses compared to 47% among the comparison group (Table 1). A larger proportion of patients in the Photovoice group had successful treatment outcomes compared to the comparison group (93% versus 82%) (Table 1). The Photovoice group had three times the odds of successful treatment outcome compared to those who did not watch Photovoice (OR: 2.8, 95%  CI: 1.3–6.1) (Table 1).

Nearly all (99%) of the Photovoice patients converted sputum at the end of IP compared to 88% in the comparison group (Table 1). Patients who did not watch the video, on average, missed a greater number of doses during treatment (10.9 doses; 95% CI: 6.6–11.1) than patients in the intervention group (5.5 doses; 95% CI: 3.7–6.1) (Table 2).

## 4. Discussion

Several studies have described factors associated with ATT adherence but few studies describe the impact of interventions leading to behavioral changes that influence treatment outcomes [6]. This study was not a rigorous randomized controlled trial; however, these results reflect meaningful programmatic experience. Improved TB health-related education, along with efforts to reduce stigma, has been shown to improve patient health-seeking behaviours, lower treatment default rates, and bring about greater treatment completion [7, 9]. To our knowledge, this is the first time that Photovoice has been used to empower TB patients to share their personal experiences to influence ATT adherence. Having patients watch a 15-minute Photovoice video prior to initiation of ATT is a low-resource activity with the potential to improve medication adherence and long-term treatment outcomes.

## 5. Conclusion

Photovoice is a simple, inexpensive strategy that might be considered as one of Government of India's Information, Education, and Communications activities to strengthen TB treatment adherence. In combination with other interventions, a Photovoice approach could be an effective tool for reduction of treatment lost to follow-up and other unsuccessful TB treatment outcomes.

## Figures and Tables

**Table 1:** Demographic, risk, and treatment outcomes of TB patients who viewed the Photovoice video and comparison group who did not, Pune, India.

Characteristics	Photovoice		Comparison		Total	*P* value
	*n*	(%)	*n*	(%)		
Gender						
Male	87	(64.4)	77	(54.6)	164	0.096
Female	48	(35.6)	64	(45.4)	112	
Age categories (years)						
<15	17	(12.6)	18	(12.8)	35	0.148
15–25	23	(17.0)	30	(21.3)	53	
26–35	33	(24.4)	42	(29.8)	75	
36–45	31	(23.0)	33	(23.4)	64	
46–55	23	(17.0)	9	(6.4)	32	
>55	8	(5.9)	9	(6.4)	17	
HIV status						
Positive	45	(33.3)	41	(29.1)	86	0.743
Negative	66	(48.9)	74	(52.5)	140	
Unknown	24	(17.8)	26	(18.4)	50	
Type of tuberculosis						
Sputum positive pulmonary	56	(41.5)	52	(36.9)	108	0.423
Sputum negative pulmonary	31	(22.9)	28	(19.9)	59	
Extrapulmonary	48	(35.6)	61	(43.3)	109	
Total number of missed doses						
No missed doses	102	(75.6)	66	(46.8)	168	** 0.000**
1 or more missed doses	33	(24.4)	75	(53.2)	108	
Sputum at the start of initiation phase^1^						
Negative	81	(60.0)	95	(67.4)	176	0.125
Positive	54	(40.0)	46	(32.6)	100	
Sputum at the end of initiation phase^1^						
Negative	133	(98.5)	124	(87.9)	257	**0.000**
Positive	2	(1.5)	17	(12.1)	19	
Treatment outcomes						
Successful	125	(92.6)	115	(81.6)	240	**0.000**
Unsuccessful^2^	10	(7.4)	26	(18.4)	36	

^1^Antituberculosis treatment (ATT) is comprised of 2 phases: intensive phase (IP) (24 doses of isoniazid, rifampicin, pyrazinamide, and ethambutol by directly observed therapy (DOT) thrice a week on alternate days for 8 weeks) and continuation phase (CP) (54 doses of isoniazid and rifampicin given thrice a week on alternate days with at least first dose of every week being directly observed). ^2^Patients with treatment failure and patients who transferred, lost to follow-up, or died.

**Table 2:** Mean number of antituberculosis drug doses missed among TB patients who viewed Photovoice and comparison group who did not by treatment outcome.

	*N* (%)	Mean number of total missed doses	Mean difference^1^
		Mean (SD)	(95% CI)^2^
*Overall* (*N* = 276)			
Photovoice			
Successful	125 (92.6)	0.6 (1.3)	4.9 (3.7–6.1)
Unsuccessful^3^	10 (7.4)	5.5 (4.9)	
Comparison			
Successful	115 (81.6)	2.1 (3.9)	8.8 (6.6–11.1)
Unsuccessful^3^	26 (18.4)	10.9 (8.6)	

^1^Equal variances assumed. ^2^CI: confidence interval, SD: standard deviation. ^3^Patients with treatment failure and patients who transferred, lost to follow-up, or died.
